# Supplementary material for: Varying crosslinking motifs drive the mesoscale mechanics of actin-microtubule composites
Source: Sci Rep. 2019 Sep 6;9:12831. doi: 10.1038/s41598-019-49236-4 (PMC6731314; doi:10.1038/s41598-019-49236-4)
Supplement: Supplementary file 1 — Supplementary Info [file 41598_2019_49236_MOESM1_ESM.pdf]

**Varying crosslinking motifs drive the mesoscale mechanics of actin-microtubule composites**

Shea N. Ricketts[a], Madison L. Francis[a], Leila Farhadi[b], Michael J. Rust[c], Moumita Das[d], Jennifer L. Ross[b] and Rae M. Robertson-Anderson[*a]

[a]Department of Physics and Biophysics, University of San Diego, 5998 Alcala Park, San Diego, CA 92110

[b]Department of Physics, University of Massachusetts, Amherst, 666 N. Pleasant St., Amherst, MA 01003

[c]Department of Molecular Genetics and Cell Biology, University of Chicago, 900 E 57th St, Chicago, IL 60637

[d]School of Physics and Astronomy, Rochester Institute of Technology, 84 Lomb Memorial Drive, Rochester, NY 14623

*randerson@sandiego.edu

## Supporting Material

**Figure S1. Impact of transient effects on microscale response of actin-microtubule composites.**

**Figure S2. Speed dependence of force response and stiffness of actin-microtubule composites.**

**Figure S3. Mobility analysis from time-series with 62.5 ms time windows.**

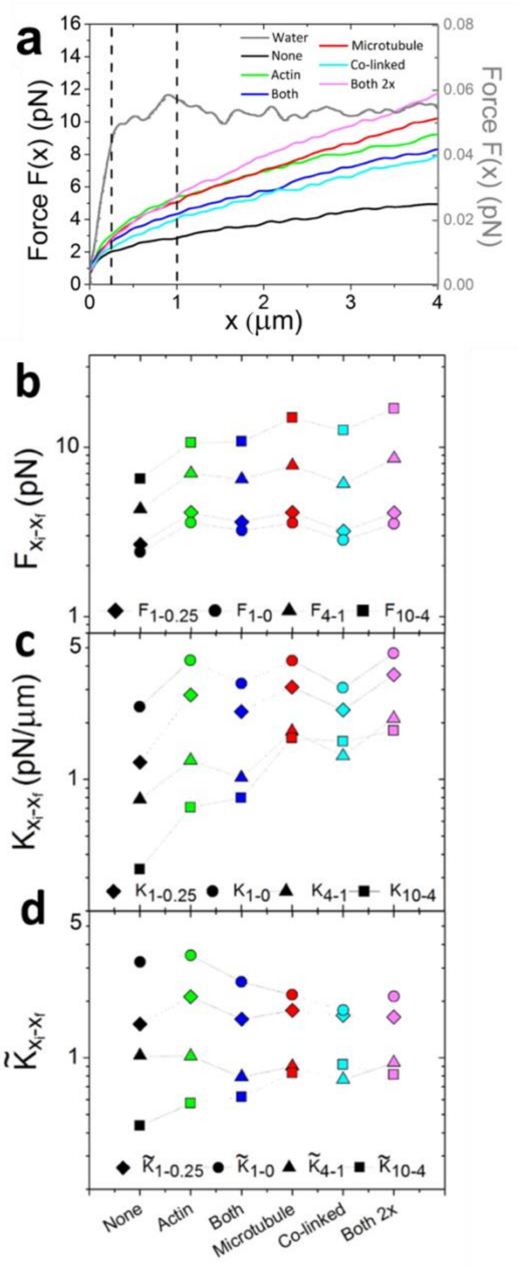


**Figure S1. Impact of transient effects on microscale response of actin-microtubule composites. (a)** Force that water exerts (grey, right-hand axis) compared to force that composites exert (black, left-hand axis) to resist a 10 μm/s strain. Dotted vertical lines denote the region over which transient effects persist (left) and the region over which the data is averaged to compute averaged quantities presented in b-d (right). (**b**) Force values from (a), averaged over different bead displacements $x_f - x_i$, denoted as $F_{xf-xi}$ (color coded as in (a)). Data shown is for $x_f - x_i$ = 1 – 0.25 $\mu$m (diamonds), 1 – 0 $\mu$m (circles), 4 – 1 $\mu$m (triangles) and 10 – 4 $\mu$m (squares). (**c**) Stiffness, $K = dF/dx$, averaged over the same displacements as in (**b**), as listed in the legend. (**d**) The dimensionless stiffness, $\widetilde{K}(x)$ = (10 μm)$d\tilde{F}(x)/dx$, averaged over the same displacements as in (**b**).

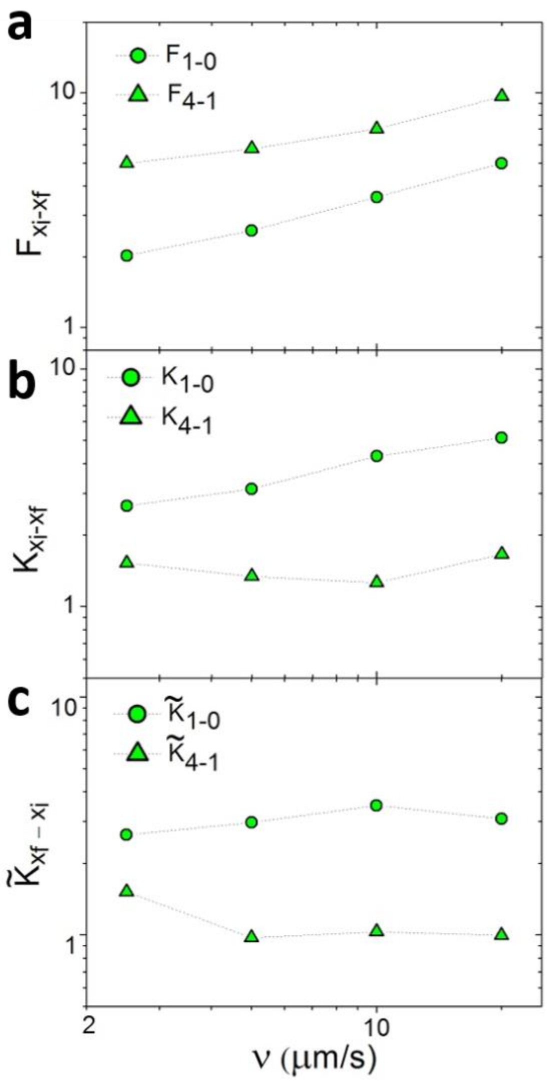


**Figure S2. Speed dependence of force response and stiffness of actin-microtubule composites.** Strain-averaged quantities presented in Fig S1 computed for strains of varying speeds $v$ for composites in which actin is crosslinked (termed *Actin* in the manuscript). Data shown is for $x_f - x_i$ = 1 – 0 $\mu$m (circles) and 4 – 1 $\mu$m (triangles). Quantities shown are (**a**) force $F$ (pN), (**b**) stiffness, $K = dF/dx$ (pN/μm), and (**c**) dimensionless stiffness $\widetilde{K}(x)$ = (10 μm)$d\widetilde{F}(x)/dx$.

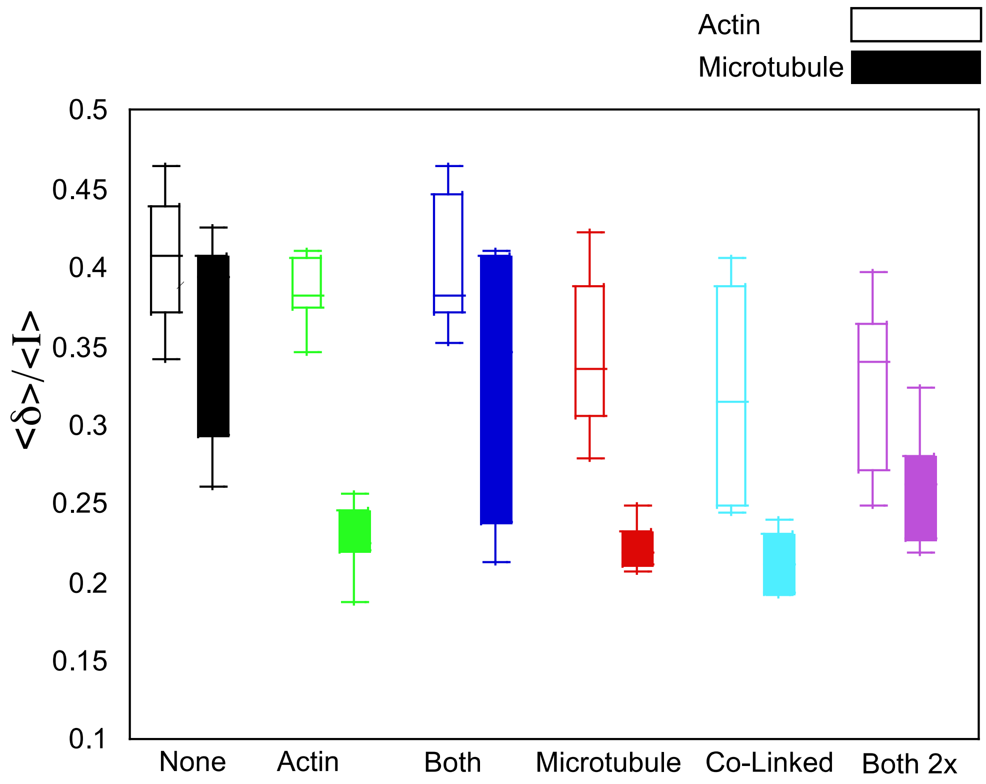


**Figure S3. Mobility analysis from time-series with 62.5 ms time windows.** Box-whisker plot of the steady-state mobility, determined by computing the average standard deviation of pixel intensities $<\delta>$ normalized by the overall average pixel intensity $<I>$ for each time-series, as described in Methods and caption of Fig 4. Here, we use a time resolution of 62.5 ms (1 frame) compared to 1 s (16 frames averaged together) shown in Fig 4. As shown, the dependence of the mobility of both actin and microtubules on crosslinking motif is robust to varying time windows used for analysis. However, for smaller time windows the mobility values and spread in the mobility distributions for both actin and microtubules are enhanced due to noise.
